# Source of Nanocellulose and Its Application in Nanocomposite Packaging Material: A Review

**DOI:** 10.3390/nano12183158

**Published:** 2022-09-12

**Authors:** Jingwen Wang, Xiaoshuai Han, Chunmei Zhang, Kunming Liu, Gaigai Duan

**Affiliations:** 1 Jiangsu Co-Innovation Center of Efficient Processing and Utilization of Forest Resources, International Innovation Center for Forest Chemicals and Materials, College of Materials Science and Engineering, Nanjing Forestry University, Nanjing 210037, China; 2 Institute of Materials Science and Devices, School of Materials Science and Engineering, Suzhou University of Science and Technology, Suzhou 215009, China; 3 Faculty of Materials Metallurgy and Chemistry, Jiangxi University of Science and Technology, Ganzhou 341000, China

**Keywords:** biodegradable, cellulose film, food packaging, green packaging, natural polymers

## Abstract

Food packaging nowadays is not only essential to preserve food from being contaminated and damaged, but also to comply with science develop and technology advances. New functional packaging materials with degradable features will become a hot spot in the future. By far, plastic is the most common packaging material, but plastic waste has caused immeasurable damage to the environment. Cellulose known as a kind of material with large output, wide range sources, and biodegradable features has gotten more and more attention. Cellulose-based materials possess better degradability compared with traditional packaging materials. With such advantages above, cellulose was gradually introduced into packaging field. It is vital to make packaging materials achieve protection, storage, transportation, market, and other functions in the circulation process. In addition, it satisfied the practical value such as convenient sale and environmental protection, reduced cost and maximized sales profit. This review introduces the cellulose resource and its application in composite packaging materials, antibacterial active packaging materials, and intelligent packaging materials. Subsequently, sustainable packaging and its improvement for packaging applications were introduced. Finally, the future challenges and possible solution were provided for future development of cellulose-based composite packaging materials.

## 1. Introduction

The improvement of people’s living standards has greatly promoted the development of packaging industry. At present, excessive packaging and packaging waste have caused great pressure to the environment. The fundamental role of packaging is to protect products, facilitate storage, transportation, and promote sales [1]. The disposal of packaging waste caused by the rise of offline shopping and online shopping has attracted widespread attention [2]. In particular, during the novel Coronavirus outbreak, the contamination of takeaway packaging waste caused by quarantine measures and other epidemic prevention measures like disposable masks and medical packaging waste have further exacerbated the contradiction between human society and environmental protection [3]. For packaging waste, degradable packaging or recyclable packaging are two common solutions. The vast majority of packaging materials on the market are non-degradable materials such as polyethylene, glycol terephthalate, polypropylene, polystyrene, high-, and low-density polyethylene. Driven by people’s awareness of environmental protection, the degradability of packaging has become one of the most important directions of new packaging in the future. Different countries have made corresponding efforts to promote packaging degradability. The European market is now taking the road leading to lightweight and green packaging [4]. The manufacturer is required to be responsible for the whole life cycle of product and form a closed-loop packaging dealing prosses. Biodegradable green packaging has become the focal point of the global packaging markets [5].

The common degradable materials contain polyester-based biomaterials synthesized from natural raw polyethers like polyhydroxyalkanoate (PHA) [6], including poly(3-hydroxybutyrate), and polylactide (PLA), and poly-β-hydroxybutyric acid (PHB) [7]. At the same time, polyglycolic acid (PGA) is an aliphatic thermoplastic linear polyester, and it can be obtained by polycondensation of glycolic acid and ring-opening polymerization of glycolate. In addition, through butadiene, cyclohexane and ring-opening polymerization can be used to synthesize polycaprolactone (PCL), which is a kind of polyester material with high biodegradability and biocompatibility [8]. Furthermore, some other biodegradable polymers such as zein, soybean, and wheat protein-based materials, can be obtained through protein-like reactions, catalyzed by proteases, to form a substance similar to a high molecular protein. Self-assemblies of lipid molecules were obtained naturally or by vesicle preparation, solvent injection, vortex dispersion, etc. to achieve lipid-based material like waxes, phospholipid, glycolipids, steroids, and terpenes [9]. They were naturally acquired or acquired by using synthetic biology method to design and synthesize high performance protein biomass materials like collagen, gelatin, and silk-based biomaterials. In addition, polysaccharide-based materials extracted from red seaweed like agarose, cephalopod intestines, shrimp, crab body surface extracted chitin and chitosan, linear macromolecular hyaluronan polysaccharide formed by disaccharide polymerization, and alginate extracted from algae material, through high temperature and low-pressure polymerization dextran with polymerization degree in the range of 200–2000, cellulose, and its derivatives [10]. Among the degradable materials, cellulosic composite packaging materials have attracted a wide amount of attention with its unique advantages. With wide sources and high yield, cellulosic composite materials make it well-known in food product packaging materials. Along with the development in nanotechnologies and its application in food packaging, currently nanofillers are used to improve the properties of polymer materials in composites. Numerous studies applied nano particles to improve packaging properties due to their excellent properties [11,12,13,14], such as mechanical and thermal properties (ZrO_2_ and SiO_2_), antibacterial properties (Silver nano particles), UV protection (ZnO), water vapor permeability (boron nitride), etc. Various environmentally friendly film composite materials are widely used in the packaging industry with inorganic salts as additives. It is reported that polyvinyl alcohol film incorporated with CaCO_3_ can improve mechanical and thermal properties of the film [15]. In addition, CaCO_3_ were added into starch-based film to improve oxygen barrier, tensile strength, and thermal stability [16]. At the same time, adding selenium nanoparticles to packaging has been reported to inhibit the reaction rate of microbial growth and reduce the use of preservatives [17]. In this regard, we need to have an overall view of nanocellulose. In packaging field, cellulose derived packaging can be broadly divided into three categories like cellulose nanocrystal, cellulose nanofiber, and bacteria cellulose nanofiber. In addition, the common composite processing methods can also be roughly introduced the following several types such as casting-evaporation processing, sol-gel processing, and other processing methods [18].

**Figure 1:**
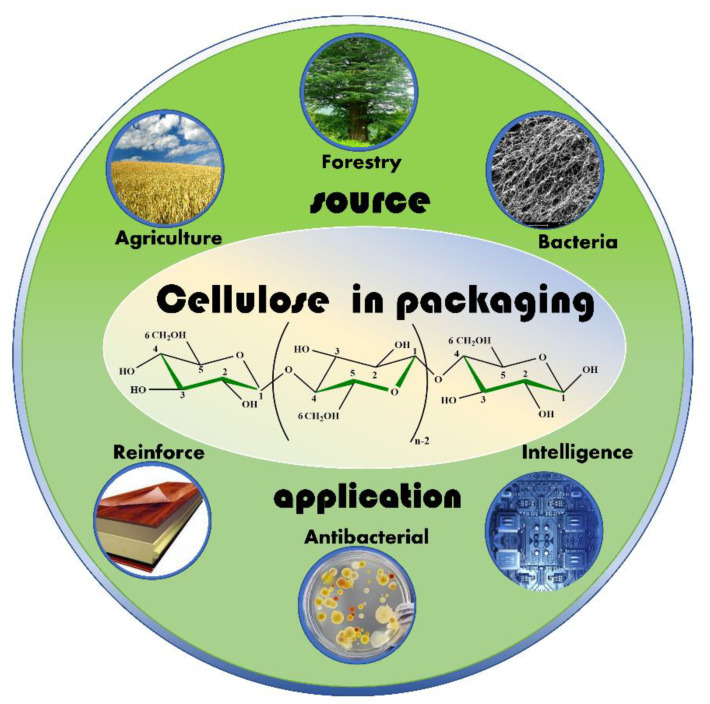
An overview of cellulose film for food packaging application.

Figure 1 provides an overview of cellulose resources, cellulose molecular structure, and its application in composite reinforcement, antibacterial ability, and intelligent packaging materials. It is of great significance to future research and development. This review contains the source of cellulose nanofibers, its preparation, and several applications in the packaging fields. With varying attention in environment protection, cellulose will be widely used in the packaging industry in the future, including retail packaging, packaging containers, and cushioning packaging. Related experiments show that the lamination process can effectively reduce the water permeability of cellulose composite, and greatly improve the water resistance and oxygen resistance of the film. Biobased nanocomposites derived materials are going to be a new development in this direction.

## 2. Sources of Cellulose

**Figure 2:**
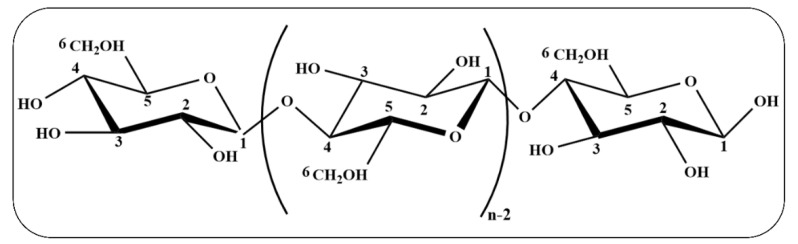
Molecular structure of cellulose.

In 1983, Herrick et al. were the first to successfully isolate cellulose microfibers around the world. Cellulose is a high molecular weight homopolymer [19]. It is now playing the focal role of skeleton in plants within a certain degree of orientation [20] (Figure 2). 

It is a kind of biomaterial with wide sources, low cost, degradable, and renewable features. The particle types of cellulose include wood fiber and plant fiber, microcrystalline cellulose, microfibrillated cellulose, nanofibrilated cellulose, cellulose nanocrystals, tunicate cellulose nanocrystals, algae cellulose particles, and bacterial cellulose particles [21]. Furthermore, the properties of nanocellulose mainly include mechanical properties, thermal properties, liquid crystallinity, rheological properties [22], and optical properties [23]. It is worth noting that cellulose plays a vital role in supporting the plant cell wall and improves the mechanical properties of plants [24]. One of the focal advantages using this biomaterial is that there are various sources of cellulose. Some of the natural cellulose comes from plants and some are produced by bacteria [25]. Cellulose microfiber isolated from different functional cells of the same plant have the same characteristics [26]. Scientists have focused on isolating cellulose from all possible crops. Commonly, cellulose from grain straws to isolate nanocrystals have a huge potential in food packaging fields. It is reported that the cellulose nanocrystals were separated from wheat straw by the acid hydrolysis method, and the carboxymethyl cellulose composite membrane was prepared by different modification treatment. The modified composite tensile strength increased by 45.7%, and the water vapor permeability decreased by 26.3%. Because of its degradability, superior mechanics, and water vapor permeability, this kind of modified cellulose nanocrystal composite material has the potential to be used in the packaging field [27]. However, every year, a large amount of forestry and agricultural waste remains due to industrial production. These untreated and underutilized byproducts contain high amounts of cellulose. With the increased production of industrial, agricultural, and forestry wastes, the accumulation of agricultural and forestry residues disposed by landfill or incineration [28], these treatments not only cause a low utilization rate of resources, but also become a burden on the environment. Therefore, this part of residues must utilize reasonably in order to reduce environmental pressure and satisfied green packaging requirements. All in all, residue cellulose is a promising develop direction [29]. Cellulose is the skeletal material that makes up the cell wall of plants, with both crystalline and amorphous regions. The crystal area is orderly, and the amorphous area is irregular. The hemicellulose and lignin in the material can be removed by pretreatment to retain the cellulose as a skeleton material, then through oxidation treatment, acid treatment, alkali treatment combined with mechanical treatment, nanocellulose can be further obtained, such as cellulose nanocrystalline and cellulose nanofibrils. One of the focal advantages using this biomaterial is that there are various sources of cellulose. There are three main sources of cellulose: plant cellulose, agricultural cellulose, and bacterial cellulose. Cellulose microfiber isolated from different functional cells of the same plant has the same characteristics. Scientists have focused on isolating cellulose from all possible plants.

### 2.1. Agricultural Derived Cellulose

The disposal of agricultural waste has always been a troubling problem. With the social advocacy of the circular economy and people’s advocacy of recycling agricultural waste resources, the rational utilization of agricultural waste can avoid environmental pollution to a certain extent [30]. Nowadays, Taiwan has revised relevant laws and regulations to improve the efficiency of agricultural waste recycling [31]. Agricultural waste has always been an important source of cellulose, and cellulose can be recovered from agricultural waste through a series of processes. In this way, agriculture waste not only creates a new renewable resource, but also maintains a great degradability to the environment. Various agricultural wastes, such as oat husk [32], sisal [33], cocoa pod husk [34], pineapple peel [35], and leaf [36], have been studied as nanocellulose resources. Straw, rice stalks, and wheat stalks are a wide range of fiber sources [37]. Cellulose can be isolated from agricultural waste operating with chemical treatments, such as alkaline treatment, bleaching, and acid hydrolysis [38]. The most common example should be sugarcane. After sugar and alcohol isolation, sugarcane bagasse becomes the main source of cellulose, using ultrasonic irradiation and various concentrations of alkali and alkaline peroxide to extract cellulose from agricultural residues [39]. Different parts of cellulose employ different extraction methods. Agricultural waste includes cellulose, hemicellulose, lignin, dietary fiber, sugar, and other components. The natural fiber part of the agriculture plant exhibits a huge potential to be the sources of cellulose. The roots [40], stems [41], leaves [42], pomace [43], and peel of plants [44] are rich in cellulose [45]. Usually, the fiber extraction process is designed differently according to different raw materials. The methods include acid [46], alkali [47], mechanical treatment, and enzyme-assisted [48] mechanical treatment [49]. Reaction time and raw material ratio have great influence on the yield, morphology, and chemical structure of cellulose. Cellulose extracted from agricultural waste has become an important research topic [50]. Table 1 illustrates cellulose extraction from different agriculture species.

### 2.2. Forestry Derived Cellulose

Forestry is a kind of sustainable natural resources. Today, with the advocacy of green and sustainable development, green chemistry and its influence in various industries are extensive and far-reaching [59]. Every country began to pay attention to forestry. Indonesia made efforts to expand forestry cultivation and co-manage. The climate environment, forestry planting space, and adaptability of forestry species were explored—from the government, society, the public, and other aspects to achieve a mixed management and protection mode of forestry [60]. Efforts in Europe were made to improve productivity, increase the innovation of forestry planting technology, maintain the sustainable development of forestry, and maintain the recovery of forestry [61]. Attach importance to the overall development of the private forestry industry through establishing a scientific forestry advisory system to provide technical knowledge and advisory services to private forestry [62]. Through the rational allocation and scientific management of forestry resources to cope with climate and environmental changes, forest species habitat limitations, and other resources and environmental problems give full play to the ecological potential of the forest [63]. These have helped to green the environment and improve the economy. Australia manages and invests in forestry to industrialize and commercialize it. Facts have proved that the commercialization of forestry has greater economic benefits for the whole society. It shows the great potential of forestry in social development. Forestry is also an important source of cellulose. Current research has extracted cellulose from different tree species. Table 2 illustrates cellulose extraction from different tree species. It shows the great potential in sustainable society development. Forestry is also an important source of cellulose. Microcellulose can be extracted from cellulose at a microscale. It also can be divided into microfibrillar cellulose and microcrystalline cellulose according to its structure. Nanocellulose with a diameter less than 100 nm can also be obtained from cellulose [64]. Current research has extracted cellulose from different tree species. At present, there are oxidation treatment, acid treatment, and biological treatment methods for wood-based CNCs and CNF preparation. Compared with cellulose, nanocellulose is a linear polysaccharide in the nanometer scale. The hydroxyl groups are mainly located on C2, C3, and C6. The crystalline region and the amorphous region are ordered structures, and the molecules interact with each other through hydrogen bonds and van der Waals forces [65]. Nanocellulose has good biodegradability and renewability, in order to improve the processing properties of cellulose nanomaterials and facilitate their functionalization, surface modification is usually required. The common surface modification mainly includes physical and chemical modification. Physical modification methods mainly include surface adsorption modification, plasma treatment, and ion beam treatment. Chemical modification methods mainly include etherification, grafting, sulfonation, silylation, trans-esterification, tempo oxidation, amidation, etc. [66]. Zhao et al. reported an acid treatment for extracting cellulose nanocrystalline from poplar. In the process of strong acid hydrolysis, hydronium ions cause hydrolysis and cleavage of glycosidic bonds to produce cellulose crystals. Cellulose nanocrystals were successfully prepared from poplar cellulose. After pretreatment, lignin and hemicellulose of poplar were removed, and cellulose skeleton was retained. The cellulose was hydrolyzed in 64 wt% sulfuric acid for 30 min at 45 °C. The machine was then washed and centrifuged three times to collect the precipitate. The precipitate was dialyzed and homogenized under high pressure. The suspension was evaporated and condensed to 0.6 wt%, then diluted to 0.8 wt% to 1 wt% and ground to milky white. In this way, nanocellulose suspension was successfully prepared [67]. Kang et al. reported an environmentally friendly method to prepare CNCs. Cellulose nanocrystals were prepared by ball milling and centrifugation. This preparation method has little environmental pollution but a low yield of CNCs. Zhu et al. mixing eucalyptus cellulose with deep eutectic solution (DES) through heating and stirring, washing, and high-pressure homogenization successfully prepared CNFs. The above are several commonly used preparation methods of wood based nanocellulose. This cellulose can be used in packaging films, coatings, and additives. In a word, forestry derived cellulose is an essential part of cellulose resource.

### 2.3. Bacteria Derived Cellulose

Bacteria cellulose is a new type of cellulose recently. It is biosynthesized by bacteria called *Acetobacter xylinum* [74]. Unlike tradition plants derived cellulose fibers, bacterial cellulose possesses a three-dimensional network structure, high biocompatibility, and excellent water holding capacity. At the same time, bacterial cellulose has excellent physicochemical properties, including high tensile strength, crystallinity, high porosity, high specific surface area, and low water release rate. Water molecules are connected by hydrogen bonds between bacterial cellulose [75]. Currently, bacteria cellulose is used in the food field [76], wound dressing in medical field, etc. [77]. It is formed like ultra-fine cellulose network ribbon [78]. The production mode of bacterial cellulose has a great influence on its structure and properties, so the study on the production and conditions of bacterial cellulose has become an important direction in the food industry, composite material, enzymatic stability, and so on [79].

### 2.4. Carboxymethyl Cellulose

Carboxymethyl cellulose is a polysaccharide obtained by cellulose etherification reaction. The main reaction to prepare carboxymethyl cellulose contains two main steps: (i) alkaline treatment, (ii) etherification. Through alkalization reaction, the hydroxyl group on the cellulose pyran glucose chain is activated to form alkali cellulose, then the activated alkali cellulose is etherized, and the carboxymethyl group is combined with the activated hydroxyl group to form carboxymethyl cellulose [80]. At present, CMC as a cellulose derived ether material is widely used in wound dressing, drug delivery, composite film [81], paper industry, food preservation coating, and other relevant fields. Carboxymethyl cellulose can be widely used in food packaging because of its good film-forming ability and no toxicity property. Currently, the research on the food packaging application of carboxymethyl cellulose mainly focuses on functional composite film, intelligent response packaging, functional coating packaging, and edible packaging. By composite with antibacterial components, such as Chinese chive [82], silver nanoparticles [83], and carboxymethyl cellulose film can achieve antibacterial properties; in addition, composite with poly vinyl alcohol/aloe can produce an active packaging film [84]. Through integrating with various functional material, combining with blending, vacuum filtration, and other membrane processes, carboxymethyl cellulose can successfully produce different functional material, which demonstrates a huge potential in functional packaging. In addition, by crosslinking with fumaric acid, carboxymethyl cellulose can exhibit pH responsive property, which can be used in pH responsive packaging to detect food freshness [85]. Carboxymethyl cellulose has the characteristics of non-toxic and degradability. Thus, using carboxymethyl cellulose immobilized silver nanoparticles as a degradable coating for food wrapping paper can greatly enhance the antibacterial performance of packaging and prolong shelf life [86]. Hence, functional coating is another essential application of carboxymethyl cellulose in packaging. According to the present report, carboxymethyl cellulose combined with other edible materials such as corn starch, lactobacillus, combined with the casting process of the film, can be used to prepare edible film. This film is an important alternative to non-degradable plastic products [87]. With the trends of consumer pursuit, non-toxic environmental protection, degradable, natural products, and carboxymethyl cellulose composite packaging products are expected to be in the field of food packaging.

## 3. Application in Food Packaging

There are many factors that can cause food deterioration. Environmental temperature, humidity, and weather conditions are the main factors. Furthermore, natural factors in the process of product transportation have a huge influence. The loading and unloading, transshipment, receipt, and delivery in the circulation of products greatly affect the quality of the product. Cellulose-derived material has been paid much attention in composite fields due to its wide sources, non-toxicity, light weight, and high hygroscopicity features. In addition to the simple composite materials [88], the research on cellulose-related thin films with special functions such as ultra-thin film [89], electrostatic self-assembled thin film [89], adsorption film [90], hydrophobic film [91], and other complex nano-reinforced films are also emerging. In external conditions such as temperature and humidity environment, cellulose’s internal structure can be easily damaged. This caused the decline of barrier property. Therefore, it is usually combined with other materials to make nanocellulose-based composite films.

### 3.1. Application in Composite Reinforced Packaging

Composite material generally refers to two-phase material. Compared with common material, composite material has more superior properties. Cellulose has many excellent properties such as degradability, nontoxic, and accessibility. Therefore, packaging material is becoming more and more interested in cellulose composite material. In general, the distribution and dispersion of cellulose fiber in composites will have a certain influence on the properties of composites such as particle size, length, and aspect ratio [92]. The common reinforcement methods are blending, adding additives, and surface modification [93]. Thus, the fiber can obtain both inherent flexibility and mechanical properties. The common nanocellulose composites mainly include polylactide based nanocellulose composites, poly-hydroxybutyrate based nanocellulose composites and starch based nanocellulose composites, etc. [94]. In experiments, surface modification or chemical thermomechanical treatment are used to solve the incompatibility problem. The modification technology includes block modification and surface modification [95]. Cellulose, especially the nanocellulose used in packaging, can improve the mechanical properties of packaging, more importantly, improve the recycling performance of packaging [96]. It has been reported that the tensile strength of the composite increased by about 20% when microcrystalline cellulose was added to polyethylene in the difficult-to-recycle multilayer packaging using a two-rod mixing process. This not only improves the mechanical properties of the material but also increases its recyclability [97]. Similarly, adding cellulose nanocrystals into faba bean protein packaging film can greatly improve the mechanical properties, thermal properties, and barrier properties, which make it more advantageous in the packaging field [98]. In addition, cellulose can improve the mechanical properties of chitosan-based packaging film [99]. With the social sustainable development, cellulose will play an essential role in composite reinforcement.

#### 3.1.1. Coating Reinforcement

Cellulose is a linear polymer with many free hydroxyl groups that can participate in various reactions to make it associative. In addition, cellulose is easy to produce strong intermolecular and intramolecular hydrogen bond connections. One way of enhancing is to use cellulose as coating for material reinforcement [100].

**Figure 3:**
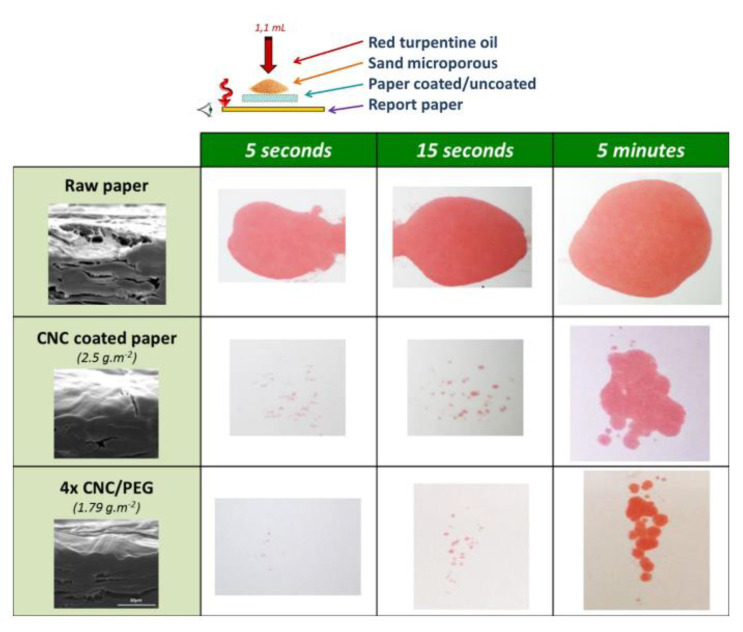
Grease test image of raw paper, CNC coated paper, and CNC/PEG coated paper.

**Figure 4:**
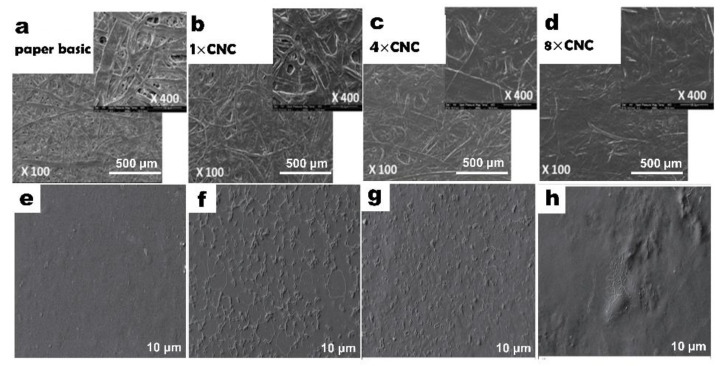
SEM image of CNC−coated Surface (**a**) raw paper; (**b**) one times CNC−coated paper; (**c**) four times CNC−coated paper; and (**d**) eight times CNC−coated paper. SEM image of CNF surface and water vapor permeability analysis; (**e**) raw paper; (**f**) 2% (*w*/*w*) additive amount of CNF; (**g**) 4% (*w*/*w*) additive amount of CNF; (**h**) 6% (*w*/*w*) additive amount of CNF.

Gicquel et al. work on cellulose nanocrystal coating. Paper based CNC coating to produce functional material, the dosage, and preparation of CNC coating are shown in the figure (Figure 3). It can be obviously observed from the SEM image that the paper coated by CNC is more compact, with fewer pores on the longitudinal section. With the increase of CNC coating times, fibers connected tightly on the surface when CNCs were coated for eight times. In addition, the fibers became more compact. In addition, the paper surface tends to be smooth, and the barrier performance is constantly enhanced (Figure 4a–d). The densified structure will make the barrier property and mechanical properties of the material improved compared with paper [101].

Fotie et al. reported a CNC coating applied on the surface of common packaging film. The experimental results showed that the optical properties of the film coated with CNC had no significant change [102]. After lamination, the mechanical properties improved, the oxygen permeability decreased, and the gas barrier property improved was well. However, the disadvantage of this method is the film would be affected in a humid environment due to the hydrophilicity of nanocellulose [102]. Using surface coating modification can effectively improve properties of the paper. Another report coated cellulose nanofiber to isolated whey protein to produce reinforced films. From the SEM image, we can identify the coating of 2% (*w*/*w*) cellulose nanofiber will make the surface of the film rough, the distance between the fibers is large, and the adhesion between the nanofiber and the film surface is low. With the increase of nanocellulose content, 8% (*w*/*w*) nanocellulose coating makes the visible fiber on the film surface decrease, and the material surface is smooth and compact. Experiments show that the film made with different cellulose concentrations possesses different properties (Figure 4e–h). Experiments exhibit the fact that, with the increase of cellulose concentration, the mechanical properties’ flexibility and transmittance decreases [103].

#### 3.1.2. Additive Reinforcement

Cellulose also can be used as an additive to the film material that produced the multiphase composite film. Cellulose derived-snail film is a material mainly composed of cellulose and snail mucus.

**Figure 5:**
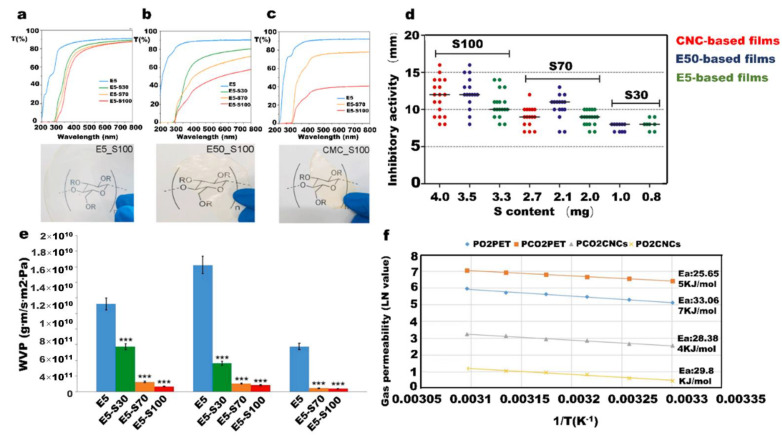
UV region of a cellulose-based snail slime film with three series of snail slime addition; (**a**) 5% (*w*/*v*) HPMC E5 polymer with 100% (*v*/*v*) snail slime film; (**b**) 5% (*w*/*v*) HPMC E50 polymer with 100% (*v*/*v*) snail slime film; (**c**) 2% (*w*/*v*) CMC Na with 100% (*v*/*v*) snail slime films and transparent images of three series film; (**d**) antimicrobial properties of the films. Tests were carried out in different disks. The diameter of each disk is 6 mm, and six strains were tested in different assays; (**e**) water vapor permeability of the film; (**f**) O_2_ and CO_2_ permeabilities in Polyethylene terephthalate coating and CNC coating.

After carboxymethyl cellulose and polymer composite, its ultraviolet 300–800 nm wavelength transmittance can reach 80%, so as to show good visible light transmittance. This composite material is highly transparent and confers a UV-screening effect (Figure 5a–c). Compared with pure polymer-based films, the cellulose-based materials have slightly higher bacterial inhibition potential (Figure 5d). Most importantly, water solubility strongly decreased compared with a single snail mucus film (Figure 5e). Furthermore, there is a report of cellulose as a filler to improve thermal properties of the film. Adding cellulose into chitosan matrix can improve the thermal stability. The thermal stability of the active film for fruit preservation has been greatly improved.

Cellulose also plays an important role in improving flexibility, transparence, and thermal stability of the film. Wang et al. reported cellulose for improving film barrier properties especially in the oxygen barrier. The material exhibits superior oxygen barrier properties (Figure 5f). This means that it is possible to use this method to produce antioxygenic packaging materials [104].

**Figure 6:**
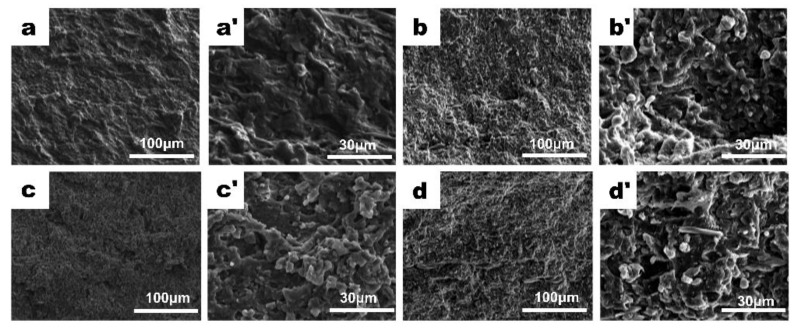
SEM images of surface topography changes with CNC contents (**a,a’**) 0 wt% cellulose nanocrystal contents; (**b**,**b’**) 0.3 wt% cellulose nanocrystal contents; (**c**,**c’**) 0.7 wt% cellulose nanocrystal contents; (**d**,**d’**) 1.0 wt% cellulose nanocrystal contents.

Jiang et al. have successfully fabricated a composite reinforced film with a high barrier to oxygen. Propylene carbonate with high oxygen resistance was used as the raw material and added poly(3-hydroxybutyrate) to increase the degradability and compatibility of the film. Results suggest that the addition of cellulose nanocrystals improves the oxygen resistance and impact strength of the composites; however, the surface area is large, which has little effect on the complex viscosity. The SEM image shows that the surface morphology changes with the increase of cellulose nanocrystalline content. At 0.7 wt% cellulose nanocrystalline addition, the composite has the densest surface and the smallest pores, so 0.7 wt% is the best addition content of cellulose nanocrystalline (Figure 6) of the films [105].

#### 3.1.3. Substrate Reinforcement

Cellulose as a substrate for a variety of specific function material is also a major application. Cellulose and lipids were used as the substrate to produce hydrophobic films. Balasubramaniam et al. reported saturated fatty acids and stearic acids to improve the hydrophobicity of the cellulose nanofiber film, making it a better hydrophobicity packaging under the same mechanical behavior [106]. Rehim et al. studied on polymethylmethacrylate (PMMA)/cellulose nanocrystal composite film. A kind of nanocomposite film was developed by blending polymethylmethacrylate (PMMA) with cellulose nanocrystals, then the water blocking performance is improved [107]. The film made by blending cellulose nanocrystals and polyvinyl alcohol carboxymethyl can improve the tensile properties of the film due to the role of cellulose. Meanwhile, cellulose as substrate is degradable in most cases. Compared with plastic, it has great advantages in environmental protection and green packaging [108]. Enhancing the mechanical properties of the film is also a major application in cellulose. Common green blends to enhance mechanical properties are cellulose whiskers [109], wheat gluten [110], etc.

#### 3.1.4. Adjuvant Reinforcement

Nowadays, cellulose has been widely used as an adjuvant in the field of improving degradability, smoothness of membrane surface, edible, water vapor barrier, mechanical properties, and antibacterial properties.

**Figure 7:**
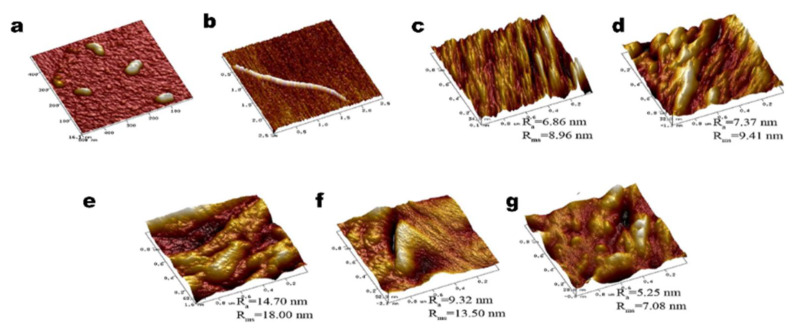
AFM images of nanocrystal cellulose/EUG composite surface morphologies (**a**) nanocrystal cellulose; (**b**) eucommia ulmoides gum; (**c**) control film; (**d**) 2% additive amount of nanocrystal cellulose; (**e**) 4% additive amount of nanocrystal cellulose; (**f**) 6% additive amount of nanocrystal cellulose; and (**g**) 8% additive amount of nanocrystal cellulose.

Sun et al. add different concentrate nano-cellulose into gutta-percha to prepare the nano-composite membrane. According to AFM observation, when cellulose nanocrystalline is used as an adjuvant material, the surface roughness is smaller, the granularity is smaller, and the average gradient is lower than that of pure cellulose nanocrystal film. With the increase of cellulose nanocrystalline content, the indexes showed a gradual upward trend (Figure 7). Eucommia ulmoides gum and cellulose are natural polymers, so the composite film has high degradability, and it can also be used in the food packaging field. The results show that the tensile stress, elongation at break, and water vapor barrier of the films are improved by adding nanocellulose [111].

Mu et al. reported a cellulose based edible film. The edible film was prepared by crosslinking gelatin with cellulose. Due to the good compatibility between gelatin and cellulose, the film has high transparency, good UV, and water vapor barrier properties [112]. Carvalho et al. added cellulose nanofiber into whey protein biopolymers. The experimental results show that the solubility of the film is reduced, and the puncture resistance and elasticity of the film are improved by adding cellulose nanofibers; it increases the rigidity of the film. However, the flexibility, and the plastic effect of the film decreased [113].

Using the degradability of cellulose to improve the degradability of the film, the biodegradable composite film was produced. This not only meets the requirements of the environment, but also meets the other functional requirements of the film [114]. Rajeswari et al. studied on cellulose based degradable films. Two kinds of non-toxic edible materials sodium alginate and carrageenan were blended with cellulose acetate to further enhance the degradability of the film and reduce the environmental pollution caused by packaging [115].

Generally speaking, other bio-based agricultural wastes rich in fibers such as sunflower seed stalks, corn stalks, and bagasse possess the potential to enhance the mechanical properties of composites [116]. In addition to the functional modification of composites by adding cellulose, some common composite preparation processes also play an important role in the functional improvement. For example, some composite films, when the composite membrane was photo-crosslinked, the water absorption of the composite membrane decreased, and the mechanical properties improved [117]. With the development of composite technology, the properties of cellulose composites are enhanced with the introduction of various new composite methods.

### 3.2. Application in Antimicrobial Packaging

Cellulose antibacterial film with green degradation, a wide range of sources, and other advantages have a higher status in antibacterial film. Scientists extracted antimicrobial ingredients from a large number of natural materials, antimicrobial nanoparticles, and synthetic antimicrobial agents. These were compounded with cellulose substrates to test their antimicrobial properties through a series of experiments. At present, a lot of progress has been made in cellulose-based antimicrobial membranes.

#### 3.2.1. Synthetic Antimicrobial Agent

**Figure 8:**
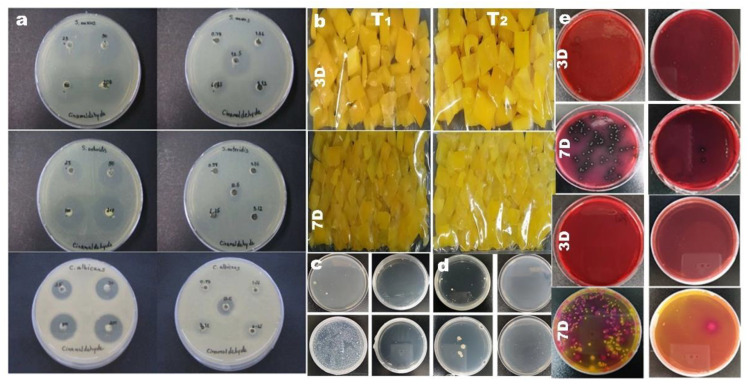
(**a**) Pictures of the bacteriostasis of cinnamaldehyde (0.78–200 μL/mL) against *Staphylococcus aureus*, *Salmonella enteritidis*, and *Candida albicans*; (**b**) 0.5% cellulose nano whisker, 3% sodium alginate and 5 mM CuO nano particles produced antibacterial polymeric film to test the freshness of pepper (contrast 3 days with 7 days); (**c**) microflora at different time intervals; (**d**) the total number of bacteria; (**e**) the total number of *Listeria* spp.(contrast 3 days with 7 days); (**f**) the total number of *Salmonella* spp.

Sanla-Ead et al. reported that cinnamaldehyde and eugenol-incorporated cellulose film have excellent antimicrobial properties. The excellent inhibitory effect of cinnamaldehyde on *Staphylococcus aureus*, Enteritidis, and *Candida albicans* has been observed (Figure 8a). Through various antimicrobial tests, they found that cinnamaldehyde and eugenol exhibit great inhibition of food pathogenic and spoilage microorganisms [118].

The test found that the mechanical properties of the composite films were improved to some extent [119]. At the present stage of the study, food film can compound the natural antimicrobial substances like capsaicin [120], gallotannin, the seeds and leaves of fruits and vegetables, etc. In addition, doping antimicrobial nanoparticles to produce antimicrobial films is also one of the commonly used methods. Saravanakumar et al. successfully prepared a kind of nano-composite antibacterial film by adding sodium alginate and copper nanoparticles into the cellulose-based film for food packaging. It was observed that, with the increase of the concentration of cellulose nano-whisker, agglomeration appeared in the film. The color of the film changes with the addition of Cu nanoparticles, but its antibacterial and antioxidant properties are improved. (Figure 8b–e) [121]. Thereby, the storage time of food is prolonged, and the shelf life is increased. The cellulose type packaging film with antibacterial properties can also be doped with nano silver ions [122], nano zinc ions [123], and so on.

#### 3.2.2. Antibacterial Coating

**Figure 9:**
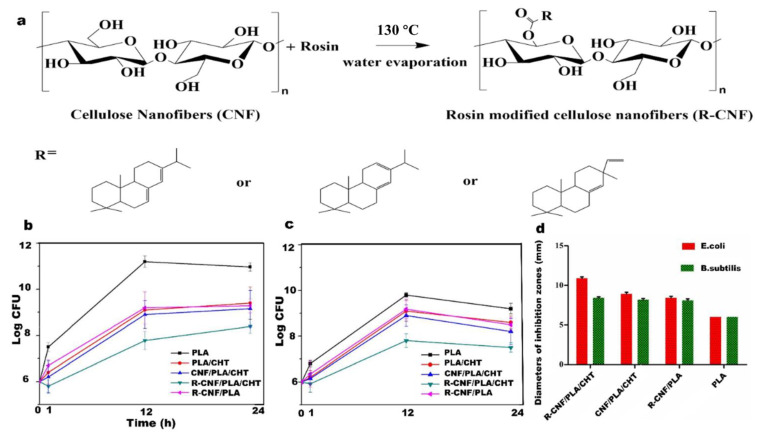
(**a**) Synthesis principle of rosin modified cellulose; (**b**) various materials on the antibacterial activity of *E. coli* (106 CFU/mL.); (**c**) various materials on the antibacterial activity of *B. subitilis* (106 CFU/mL.); (**d**) inhibition region of rosin modified cellulose nanofiber/polylactic acid/chitosan, rosin modified cellulose nanofiber/polylactic acid, polylactic acid/chitosan, and raw polylactic acid films.

The third production method is the use of antibacterial coating process. Recently, chitosan is always used as antibacterial material in packaging because of its certain antibacterial properties [124]. Niu et al. conduct research about rosin modified CNF (Figure 9a) adding polylactic acid and being coated with Chitosan to improve antimicrobial properties. Through testing, PLA/CHT based CNF reinforced film exhibits excellent antimicrobial behavior (Figure 9b–d) [125].

In addition, there are several antibacterial materials that can be used as coatings like lemongrass oil [126], thyme essential oil [127], *Laurus nobilis* essential oil [128], etc.

### 3.3. Application in Intelligent Food Packaging

With the development of people’s awareness in food freshness detection and food shelf life, consumers pay more and more attention to food safety. In the process of product transportation, temperature, humidity, external impact, and other factors are likely to have a great impact on the freshness of food. Scientists define intelligent packaging as a packaging system capable of performing intelligent functions such as detection, sensing, recording, tracking, and communication. At present, intelligent packaging gradually displays its great potential in improving food safety, the research on intelligent packaging materials has become an important direction in the packaging field [129]. Intelligent food packaging is a packaging method to improve testing and track the packaged food storage atmosphere to detect food freshness and safety when transport and selling [130]. Take meat food as an example, and fresh meat food has no ammonia substances, so the pH is generally between 5.8 and 6.2. When microorganisms cause meat corruption, the pH of ammonia substances will be greater than 6.7. At present, the time temperature indicator label (TTI) has been successfully used to detect the freshness of products in cold chain packaging. In the future, it is necessary to find independent effective and low-cost active labels and smart labels to further test the freshness of products. Ebrahimi Tirtashi et al. were dedicated to a fabricated pH indicator to test milk freshness using cellulose and chitosan-based anthocyanins of black carrot composite materials [131].

**Figure 10:**
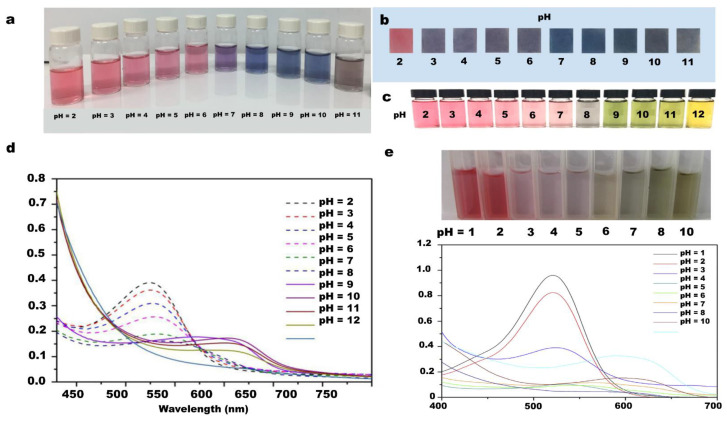
Color reaction of anthocyanins solution from black carrot at different pH conditions (pH 2–11). (**a**) color reaction of the solvent; (**b**) color reaction of indicator after immersion in solution; (**c**) color change of grape skin extraction solution at different pH conditions; (**d**) UV–Vis spectrum of grape skin extraction solution at different pH conditions; (**e**) image and UV-Vis spectrum of anthocyanin solutions in different pH conditions.

Because anthocyanin displays different color responses when placed in various pH atmospheres, significant environmental changes were observed between pH from 2 to 11. Therefore, it has the potential to be used in the detection of food freshness. (Figure 10a,b) [131].

It can be seen from the UV-Vis sprectrum that grape skin extract and anthocyanin solutions exhibit different absorption intensity to light wave at different pH. In the range of pH 2–7, grape skin extract and anthocyanin solution have the highest light absorption intensity with a wavelength of about 550 nm, and when pH 7–12, the peak light absorption intensity is about 600 nm. Further observation showed that the absorption intensity of grape skin extract decreased in a regular stepwise and isometric manner with the change of pH, while the absorption intensity of anthocyanin solution decreased sharply in a cliff-like manner when pH was 2–3. In terms of its application in intelligent packaging labels, grape skin extract has better observability and applicability. In the aspect of intelligent monitoring packaging, anthocyanins are generally selected as the best pH detection material. In the study of Qianyun Ma, the anthocyanins extracted from grape skin are compounded with tara gum/CNC. Due to the different color changes of anthocyanins under different pH conditions (Figure 10c), the changes of packaging environment can be visually displayed, and then the freshness of the product can be detected [132]. Ding et al. fabricated a pH sensor that was prepared by grafting PVA and regenerated cellulose onto acid chromotropic dye. The sensor showed different color changes when pH was 7, 10, 12. Compared with other sensors, this naked-eye detection pH sensor had fast response and high color discrimination. It had good acid and alkaline resistance, and could be used for the detection of shrimp freshness [133]. In addition, Chen et al. have developed a kind of intelligent packaging film, adding purple cabbage anthocyanins into cellulose nanofiber matrix, using anthocyanins as pH indicator. From the experiment, with the change of pH, the color changes from red to purple (Figure 10d,e). The related tests showed that the film also showed good antibacterial activity and mechanical properties [134]. In addition, anthocyanins extracted from rose [135], mulberry [136], red cabbage [137], and alizarin can also show different colors under different pH conditions. Experimental results show that the anthocyanin is compatible with different film-forming substrates. Moreover, rose green pigment has a good color reaction to meat products [138].

## 4. Degradation of Cellulose-Derived Food Packages

High biodegradability is an important advantage in cellulose-based packaging materials. Therefore, the degradation method of cellulose has become an important research direction. The essence of cellulose degradation is decreasing the degree of cellulose polymerization, and various small molecular compounds are generated under external conditions during processing. External conditions include physical, chemical, and biological processes. The degradation methods of cellulose-based materials used for packaging can be divided into three main categories: physical degradation, chemical degradation, biological degradation, and synergistic degradation. Each of the three treatments has its advantages and disadvantages. The following Table 3 lists several comparisons of advantages and disadvantages.

In general, the main factors affecting cellulose degradation rate are temperature, solution concentration, and reaction time. The thermal degradation of cellulose at different temperatures (25–300 °C) has been reported [147]. In the degradation of cellulose by calcium thiocyanate, as the amount of hydrate in calcium thiocyanate decreases, the hydrolysis rate increases; reaction time is also very important in degradation, especially in enzymatic degradation. With the denaturation of enzymes, the degradation environment pH changes and gradually stops [148].

The following Table 4 lists several degradation methods and their application directions.

### 4.1. Physical Degradation

The physical degradation of cellulose is mainly thermal degradation. Borsoi et al. reported a method for thermal degradation of cellulose. In this method, two kinds of cellulose were mechanically defibrillated, and then a certain proportion of deionized water was used to make a suspension, which was ground for 5 h, and then freeze-dried. The fiber has been observed to form nanoscale cellulose by treatment. The repeating unit and diameter of the sample polymer were significantly reduced. Similarly, Wang et al. used thermal degradation to degrade cellulose from cedar and beech wood. The cellulose was ground by ball mill for a certain time. The crystallinity of cellulose is decreased due to the cleavage of ether bond. By this thermal degradation method, cellulose was effectively degraded [160]. At the same time, lignin content affects the thermal degradation of nanocellulose. Lignin can make the composite system have better compatibility, which plays an important role in improving the polymer bonding [161].

### 4.2. Chemical Degradation

The chemical degradation of cellulose mainly includes ionic solution degradation [162], acid degradation, and alkali degradation. Zhou et al. reported a method for chemical degradation of cellulose. Cellulose is dissolved into two ionic solutions, 1-allyl-3-methylimidazole chloride and 1-ethyl-3-methylimidazole phosphate diethyl ester, respectively. It was found that cellulose was effectively degraded by experiments. Acid hydrolytic degradation of cellulose is also a common method of chemical degradation. Silica film was prepared by a spin coating method. Cellulose was hydrolyzed by exposing cellulose nanofiber film under hydrochloric acid pressure [163]. This method can visually observe the process of fiber degradation. The degree of cellulose polymerization can be reduced by acid hydrolysis of cellulose, and it can be observed that most xylan has been depolymerized by the obtained images [164]. Testva et al. reported a method of degradation of cellulose with sodium bicarbonate after acid hydrolysis pretreatment, which degrades cellulose under alkaline conditions [165]. Each of the three degradation methods has its own advantages and can meet different needs of degradation.

### 4.3. Biodegradation

Biodegradable cellulose is mainly degraded by microorganisms and enzymes. Chen et al. reported a method of microbial degradation of cellulose. Straws, leaves, and other cellulosic wastes were composted for cellulose degradation. This degradation method also provides a new idea for cellulose degradation. Similar cellulose degrading microbes are citrobacter freundii, streptomyces [166], and so on.

### 4.4. Synergistic Degradation

Li et al. reported a method for degrading cellulose by bacteria and enzymes in synergy. Under the condition of degradation of cellulose by *Clostridium thermophilus*, cellulose disaccharides inhibited the further hydrolysis of cellulose, and the presence of β-glucoside enzyme effectively alleviated the inhibition. This bio-coordinated degradation method provides a new idea for cellulose degradation. In addition, the synergistic effect can significantly increase the degradation of cellulose. Bai et al. reported a bio-functional catalytic system for the degradation of microcrystalline cellulose to 5-hmF catalyzed by CrCl_3_ and tetraalkylammonium perrhenium (1–5). This method is simple, quick, easy to operate, and it is a practical method of collaborative degradation. However, the key to improving the yield of cellulose degradation is to grasp the experimental conditions such as dosage, time, and temperature [167].

## 5. Conclusions and Future Perspectives

This article reviews the sources of cellulose and its applications in strengthening antibacterial and intelligent packaging. The extraction method of cellulose and its effect on packaging have been introduced in detail in this paper. The application of cellulose in packaging is essential for green packaging and industrial packaging production.

In the future, the development of packaging will mainly focus on the improvement of new technology [168], preservation, and environmental protection. On this basis, there will be higher performance expectations for packaging. The development of new packaging with many functions, such as high barriers, high mechanical properties, anti-bacterial, anti-oxygen and freshness detection, will become the trend of packaging development in the future. With further research on the functional modification of cellulose, cellulose-, especially the nanocellulose-, based packaging will be widely used in hydrophobic packaging, modified atmosphere packaging, high barrier film, cooking film, antistatic film, and vacuum packaging. In the field of composite flexible packaging, the common production process is extrusion composite, coating composite, solvent free composite, etc. However, the cellulose derived packaging materials are still facing the problem of the influences by the environment. This means that nature cellulose has difficulty satisfying the requirements of industrial packaging and faces great challenges in composite flexible packaging. Since most cellulose based packaging materials are composite materials, it is necessary to optimize the process of polymerization degree regulation, surface functional modification and material compatibility. The modification of agricultural cellulose to enhance cellulose rigidity and light transmittance is expected to replace polystyrene in blister packaging. By enhancing the toughness of cellulose, it also can replace polyethylene in body-fitting packaging. In addition, through modifying the thermal stability of cellulose, it is expected to be used in shrink packaging. In addition, a large number of reagents used in the cellulose and nanocellulose extraction process will have a great impact on environment. From the perspective of being green and sustainable, the process should be optimized to avoid the burden on the environment. Sustainable packaging can be further promoted by collecting waste materials for efficient conversion and reducing the use of harmful chemicals. Only in this way can we create opportunities to truly achieve sustainable goals.

## Figures and Tables

**Table 1:** Illustration of cellulose extraction from different agriculture species.

Agricultural Species	Pretreatment	Treatment	Experimental Method	Ref.
Sweet potato vine	Cut sweet potato vine (30 g) about 3 mm in length	Acid and alkali treatment	NaClO_2_ (10 g/L), acetic acid (2 mL/L) bleaching (70 °C) for 1 h, added acetic acid (2 mL) every hour three times; 6 wt% KOH solution (80 °C) for 3 h; washed by water;	[51]
Onion and garlic	Onion, garlic stalks and peels washed, dried, then pulverize	Acid and alkali treatment	The mixed solution of toluene and ethanol (2:1) was reflux for 6 h; 0.7% (*w*/*v*) NaClO_2_, 100 °C, 2 h, fiber to solution mass ratio 1:50;	[52]
Orange mesocarp	Cut the orange mesocarp into pieces, drying under 105 °C to completely dry, then pulverize	Acid and alkali treatment	2.7% (*w*/*w*) NaOH for 180 min, 5.3% (*w*/*w*) NaClO_2_, H_2_O_2_ 9.3% (*w*/*w*) continuous bleaching;	[53]
Buckwheat and rice	Immersed in acetone solution at room temperature for 24 h	Acid and alkali treatment	NaClO_2_ under pH 4–5, 75 °C bleaching four times; 4% (*w*/*w*) NaOH solution treated for 1 h;	[54]
Rice husk and coffee husk	Pulverize residue into averaged particle size 2–3 mm	Acid and alkali treatment	4 wt% NaOH mass of solid: mass of solution 1:20 stirred for 3 h; 1.7 wt% NaClO_2_ with mass of solid: mass of solution 1:20 for 4 h	[55]
Rice and oat husks	Wash the residue and dry for 24 h with temperature of 50 °C;	Acid and alkali treatment	NaOH (27 g/L) acetic acid (75 g/L) and 1 L distilled water, 90 °C for 4 h;	[56]
Arecanut husk	Immersed in toluene: ethanol (*v*/*v*) = 2:1, 48 h with temperature 50 °C; wash with boiling water and dry; cut into 6 mm on average;	Acid and alkali treatment	5% NaOH 50 °C for 4 h; 3.5 M NaClO_2_ for bleaching; milling into pulp treated with alkali again and then 5 M NaClO_2_ bleaching again;	[57]
Rice bran	0.3% α-amylase treated for 30 min with 95 °C, centrifuge (10 min)	Enzyme alkali and acid treatment	4% NaOH treatment for 75 min; washed with water, ethanol, acetone 2 times; 0.1 M HCl, 10% (*w*/*v*) NaClO_2_ 12 h at a temperature of 25 °C	[58]

**Table 2:** Comparison of cellulose extraction from different tree species.

Tree Species	Pretreatment	Treatment	Experimental Method	Ref.
Eucalyptus	Saw into powder	Alkali treatment	1% NaOH and NaHClO_3_ bleaching	[68,69]
Doum	Grind	Alkali treatment	4% NaOH and 1.7% NaClO_2_ bleaching	[70]
Banyan root	Mechanical decorticate	Alkali treatment	5% (*w*/*v*) NaOH and dilute	[71]
Mesquite	Wash crash grind and mill	Acid hydrolysis and alkali treatment	20% NaOH 5% Na_2_S 0.04% anthraquinone and 5% NaClO_2_	[72]
*Agave gigantea*	Wash, soak in boiling water, remove outer layer, dried fiber and ultrafine grinding	Alkali treatment	5% (*w*/*v*) NaOH for 2 h at 80 °C, washed, dried 60 °C for 14 h,	[73]

**Table 3:** Compared advantages and disadvantages of three main degradations.

Degradation Type	Commonly Used Reagent	Advantages	Disadvantages	Ref.
Chemical degradation	HCl; acetylsalicylic acid; Calcium Thiocyanate; 1-butyl-3-methyl imidazole chloride and WCl_6_;	Degradation rate can reach more than 80%; The reaction is relatively stable; short reaction time and strict reaction conditions;	Serious pollution to the environment; strict reaction conditions;	[139,140,141,142]
Physical degradation	Thermal degradation; Photodegradation; Electron laser;	Less pollution to the environment; the reaction is controllable;	High experiment cost; Less degradation quantity;	[143,144]
Biological degradation	Cellulase; Pectinase; Hemicellulose; Rhizobium sp.; Aeromonas caviae; Serratia sp.; Lytic polysaccharide monooxygenases;	No pollution to the environment; suitable for the degradation of large amounts of cellulose;	The composting of bacterial flora takes a long time; the enzyme activity was unstable so the degradation yields are unstable; Cellulase from different sources varies greatly in structure and function;	[145,146]

**Table 4:** Degradation methods and their application.

Degradation Type	Treatment	Experimental Method	Ref.
Mechanical, enzymatic degradation	Ultrasound, cellulose	Ultrasound with 38 KHz; 50 mg cellulase per liter; pH range 4.8–5.5; 0.1 mol NaOH or 0.1 mol HCl and 2 g powdered crystalline cellulose.	[149]
Mechanical, thermal degradation	Defibrillation, lyophilization degradation	Defibrillated in a super mass colloid with 4.5 wt% distilled water for 5 h at a speed of 1500 rpm; frozen at −80 °C; lyophilized at −40 °C for 3 days	[150]
Mechanical, thermal degradation	Freeze-dried, air-dried dehydration, themo-oxidation	pH = 4.518 prior to freeze drying. 15 mg sample under air and nitrogen at heating rates of 1, 3, 5, 10, 15, 20, 30, 40, 50, 75, and 100 °C/min between 25 and 800 °C	[151]
Photocatalytic degradation	Nano-Graphene Oxide-Type	CA (30 mg/mL) and 2.5 wt% carbon dots in 5 mL acetone; ultrasonic bath dried in an oven at 80 °C; 50 °C with 370 nm UV light irradiation	[152]
Chemical, enzyme degradation	Chitinase, cellobiohydrolase	Using 100 mg of each substrate; 5 mg of the different substrate; pH = 6 with 20 μL of 10 mM gallic acid and 5 μL enzyme; 25 °C, 900 rmp in the dark, covered with an O_2_ permeable foil for 145 h.	
Bacteria, enzyme degradation	Beta-glucosidases of cytophaga hutchinsonii	*E. coli* strains cultivated in medium supplemented with 0.2% (*wt*/*vol*) glucose; 0.2% (*wt*/*vol*) cellbiose; cells were grown with 0.2% (*wt*/*vol*) glucose at 30 °C with antibiotics.	[153]
Chemical degradation	Glucose; DMSO; copper (II) ethylene diamine solution	4 wt% cellulose with dissolution temperatures were varied from 90, 100, 110, 120, 130 °C within 72 h; then dried.	[154]
Thermal degradation	Drying; alkali; heat	10 mg of extracted sample was heated from 25 to 600 °C at different heating rate of 3 °C/min, 7 °C/min, 11 °C/min, and 15 °C/min	[155]
Hydrothermal degradation	Hydrothermal; acid	3.5 g NaOH, 0.3 g ZnO and 46.2 g deionized water with a solvent (1.75 mol/L NaOH/0.074 mol/L ZnO) stirred; take 2 mg of cellulose solution into 100 mL stainless steel autoclave.	[156]
Alkaline hydrothermal degradation	Hydrothermal; alkaline	3 g NaOH, 1 g urea, and 45 g deionized water were added into a flask and stirring at 4 °C for 2 h. 0.2–2.5 microcrystalline cellulose was added under stirring. −20 °C for 12 h. They were then thawed and added into an autoclave.	[157,158]
Bacterial degradation	*Citrobacter freundii*	Microcrystalline cellulose 5 g were added into minimal medium consisting of 2 g/L Na_2_HPO_4_, 1.32 g/L KH_2_PO_4_, 1 g/L NH_4_Cl, and *Citrobacter freundii*, etc.	[159]

## Data Availability

Not applicable.
